# PARP Inhibition with 3-Aminobenzimide Attenuates Behavioral, Cardiovascular, and Neuroinflammatory Effects of Chronic Stress

**DOI:** 10.64898/2026.04.28.721400

**Published:** 2026-05-01

**Authors:** Liza J. Wills, Hui Wang-Heaton, Aaron J. Polichnowski, Kristy L. Thomas, Benjamin E. Jewett, Seth Jewett, Grayson Aldridge, Gregory A. Ordway, Russell W. Brown, Michelle J. Chandley

**Affiliations:** a Department of Biomedical Sciences, Quillen College of Medicine, East Tennessee State University, Johnson City, TN, USA 37614

**Keywords:** major depressive disorder, PARP inhibitor, neuroinflammation, microglia, chronic stress

## Abstract

**Background:**

Major depressive disorder (MDD) affects approximately 20% of the population, with over 30% of cases demonstrating treatment resistance. Postmortem analyses have revealed increased poly (ADP-ribose) polymerase 1 (PARP-1) expression in prefrontal cortical white matter of individuals with MDD, suggesting PARP-1 as a potential therapeutic target. Chronic stress, a major risk factor for depression, affects multiple physiological domains including behavior, cardiovascular function, neuroinflammation, and gut-brain axis signaling.

**Methods:**

We conducted a comprehensive multi-system investigation of PARP inhibition effects on stress-induced pathophysiology using the social defeat stress/chronic unpredictable stress (SDS+CUS) rodent model. In the primary study, male Sprague-Dawley rats (N=32) underwent 10 days of SDS+CUS while receiving daily treatment with the PARP inhibitor 3-aminobenzamide (3-AB; 40mg/kg), selective serotonin reuptake inhibitor fluoxetine (FLX; 10mg/kg), or saline (0.9% NaCl), with non-stressed controls included. Behavioral outcomes were assessed via sucrose preference and social interaction tests. Neurobiological analyses examined PARP-1 expression, microglial morphology, and proinflammatory cytokine levels (IL-1β, TNF-α, IL-6) in relevant brain regions. In a parallel cardiovascular study, a separate cohort of stressed rats (N=8) received either saline or 3-AB treatment while hemodynamic parameters were monitored via telemetry before, during, and after stress exposure. Exploratory gut microbiome analyses were also conducted (see Supplemental Materials).

**Results:**

Saline-treated stressed rats demonstrated significantly elevated anhedonia and social avoidance compared to all other groups, while 3-AB treatment prevented these behavioral deficits. Cardiovascular monitoring revealed that stressed saline-treated rats developed significant elevations in systolic and mean blood pressure with decreased heart rate compared to baseline, whereas 3-AB treatment prevented these hemodynamic changes. Neurobiological analyses showed that FLX-treated stressed rats unexpectedly exhibited elevated PARP-1 expression in prefrontal cortical gray matter. Microglial morphological analysis revealed significantly more prolate (activated) microglia in the saline-treated stressed rats compared to all other treatment groups. Saline-treated stressed rats exhibited significantly increased hippocampal proinflammatory cytokines, with 3-AB treatment specifically normalizing TNF-α levels.

**Conclusion:**

PARP inhibition with 3-AB provides multi-system protection against chronic stress effects, preventing behavioral deficits, cardiovascular dysfunction, and neuroinflammation. These findings establish PARP-1 as a key mediator in the systemic pathophysiology of chronic stress and highlight PARP inhibition as a promising therapeutic approach for stress-related disorders with treatment-resistant features.

## Introduction

Major depressive disorder (MDD) is a leading cause of disability worldwide, with a lifetime prevalence rate of approximately 20%^1,2^. Despite the prevalence and impact of MDD, current pharmacological treatments remain inadequate, with over 30% of patients demonstrating treatment resistance^3,4^. This substantial treatment gap highlights the critical need for novel antidepressant approaches based on improved understanding of MDD pathophysiology beyond monoaminergic mechanisms.

Converging evidence indicates that neuroinflammation plays a significant role in MDD etiology^5,6^. Individuals with MDD exhibit elevated levels of inflammatory markers, including proinflammatory cytokines such as interleukin-1β (IL-1β), interleukin-6 (IL-6), and tumor necrosis factor-alpha (TNF-α) in key regions known for mood regulation such as the prefrontal cortex (PFC) and hippocampus (HPC)^7^. Additionally, the bidirectional relationship between peripheral inflammation and central neuroinflammatory processes is now well-established, with several pathways identified for immune-brain communication^8^. MDD frequently co-occurs with conditions characterized by chronic inflammation, suggesting common underlying pathological mechanisms^9,10^.

Chronic psychological stress, a major risk factor for depression, is associated with increased reactive oxygen species (ROS) production, oxidative stress, and subsequent DNA damage^11^. Microglia, the resident immune cells of the CNS, are particularly sensitive to stress signals and can become primed or activated during chronic stress, releasing proinflammatory cytokines and ROS that further exacerbate neuroinflammation^12-15^.

This creates a potential positive feedback loop between oxidative stress, microglial activation, and neuroinflammation that may contribute to the development and maintenance of depressive symptomatology.

The systemic nature of chronic stress extends beyond neurobiological changes to encompass cardiovascular dysfunction. Chronic stress exposure is associated with hypertension, altered heart rate variability, and elevated cardiovascular disease risk^16-18^. These multi-system effects suggest that effective antidepressant interventions may need to address not only central neurobiological mechanisms but also peripheral physiological dysfunction.

Poly (ADP-ribose) polymerase 1 (PARP-1), a nuclear enzyme primarily involved in DNA repair, has been implicated in this pathophysiological cascade. Under conditions of excessive oxidative stress, PARP-1 becomes overactivated, depleting cellular NAD^+^ reserves and promoting proinflammatory signaling through nuclear factor kappa B (NF-κB) co-activation^19,20^. Importantly, we have previously demonstrated that postmortem brain tissue from individuals with MDD have increased PARP-1 mRNA expression in PFC white matter compared to psychiatrically normal tissue^21^. This suggests that PARP-1 overactivation may be a key molecular link between chronic stress, neuroinflammation, and depression.

PARP inhibitors, already FDA-approved for cancer treatment, have demonstrated promise in reducing neuroinflammation in various neurological conditions^22,23^. Previous research showed that the PARP inhibitor 3-aminobenzamide (3-AB) counteracted lipopolysaccharide-induced depressive-like behaviors in mice through anti-inflammatory mechanisms^10^. Additionally, our laboratory demonstrated antidepressant-like effects of 3-AB in the forced swim test and in a combined social defeat stress/chronic unpredictable stress (SDS+CUS) paradigm^24^. More recently, a case report from our group documented rapid antidepressant and anxiolytic effects following administration of the PARP inhibitor niraparib in a patient with treatment-resistant depression^25^. These findings collectively suggest PARP inhibition may represent a novel antidepressant strategy targeting neuroinflammatory processes.

The present study aimed to comprehensively investigate the multi-system effects of 3-AB in the SDS+CUS rodent model of depression, examining behavioral, cardiovascular, and neuroinflammatory markers. We hypothesized that 3-AB would prevent stress-induced anhedonia and social avoidance while reducing neuroinflammatory markers, prevent stress-induced increases in arterial blood pressure, and preserve gut microbiome homeostasis. Through parallel behavioral and cardiovascular studies, combined with detailed neurobiological analyses, we sought to establish PARP-1 as a key mediator in the systemic pathophysiology of chronic stress and to provide evidence for PARP inhibition as a multitarget therapeutic approach for treatment-resistant depression.

## Materials and Methods

### Laboratory Animals

Adult male Sprague Dawley rats (Envigo, Indianapolis, IN), approximately 60 days old upon arrival, were used as experimental subjects ("intruders") or controls across two cohorts: a primary behavioral/molecular cohort (n = 32) and a separate cardiovascular monitoring cohort (n = 8). Male Long-Evans Hooded rats served as aggressors ("residents") in the social defeat paradigm and were pair-housed with ovariectomized female Sprague-Dawley rats. Non-stress control rats were group-housed, while stressed rats were singly housed with environmental enrichment according to NIH guidelines for the Care and Use of Animals. All animals were maintained on a 12h light/dark cycle with food and water available *ad libitum*. All procedures were approved by the East Tennessee State University Animal Care and Use Committee.

### Drugs and Solutions

3-Aminobenzamide (3-AB; 40mg/kg/day) and fluoxetine HCl (FLX; 10mg/kg/day) were obtained from Sigma-Aldrich (St. Louis, MO) and diluted in saline solution (0.9% sodium chloride). Drugs were administered daily in the morning, with 3-AB administered subcutaneously and FLX administered intraperitoneally throughout the 10-day stress protocol.

### Experimental Protocol

The stress model combined social defeat stress (SDS) and chronic unpredictable stress (CUS) over 10 consecutive days, as previously described^21,24^.The SDS paradigm was conducted every morning, in which each "intruder" rat was placed in a "resident" cage for 5 minutes or until the resident performed a 2-second pin. Behaviors observed included defensive posturing, freezing, and vocal distress from intruders, and investigative sniffing, piloerection, and attack behaviors from residents.

The CUS protocol exposed rats to various mild stressors at unpredictable times, including 30-min restraint, 1h shaking and crowding, 10-min cold water (18°C) swim, 15-min warm water (25°C) swim, and 24h tipped cage. Each stressor was administered twice over the 10-day period at various times in the afternoon and evening.

Animals were divided into 4 experimental groups: (1) SAL/Stress: stressed rats receiving saline (n = 8); (2) 3-AB/Stress: stressed rats receiving 3-AB (n = 8); (3) FLX/Stress: stressed rats receiving fluoxetine (n = 8); and (4) CTRL/No-Stress: non-stress control rats (n = 8).

### Assessment of Arterial Blood Pressure and Heart Rate

A separate cohort of male Sprague Dawley rats (n = 8) was used specifically for cardiovascular monitoring during the stress paradigm. Animals were divided into two groups: SAL/Stress (n = 4) and 3-AB/Stress (n = 4). Acetaminophen (200mg/kg, PO) was added to animals' water bottles 48 h prior to surgery and remained on home cages for 72 h post-operation as an analgesic. Radio-telemetric transmitters (HD-S10; Data Sciences International, St. Paul, MN) were used to assess arterial blood pressure and heart rate (HR). Under isoflurane anesthesia (2-3%, INH), the blood pressure catheter was inserted into the right femoral artery via an inguinal incision and advanced to the abdominal aorta. The body of the transmitter was positioned intraabdominally via a midline incision and secured to the abdominal wall with sutures. Once the incision was closed, bupivacaine (TOP) was applied to the site as a local anesthetic. Rats recovered for a minimum of 7 days prior to baseline data collection. Hemodynamic parameters including systolic blood pressure (SBP), diastolic blood pressure (DBP), mean arterial pressure (MAP), and heart rate (HR) were obtained at a sampling rate of 500 Hz using Ponemah software (Data Sciences International). Baseline recordings were collected continuously during the dark cycle (1900h-0700h) for 3 consecutive days. During the stress protocol, hemodynamic data were recorded continuously during the 5-min social defeat session and again during the animal's dark cycle. Post-stress recordings continued during the dark cycle for 3 consecutive days following protocol completion.

### Behavioral Assessments

#### Sucrose Preference Test.

The sucrose preference test was conducted on experimental days 8-10 during the first 2 hours of the dark cycle (1900h – 2100h). Rats were presented with two pre-weighed bottles, one containing water and the other containing 0.8% (w/v) sucrose solution. Sucrose preference was calculated as the percentage of sucrose solution consumed relative to total fluid consumption.

#### Social Interaction Test.

The social interaction test was conducted on experimental day 11 in a locomotor arena (91cm^3^ black plexiglass box) divided by wire mesh. The experimental rat was placed on one side of the arena for a 10-min habituation period, after which a novel aggressor rat was placed on the opposite side for a 5-min interaction test. Time spent in the interaction zone (near the wire mesh) versus the avoidance zone was recorded using ANY-maze video tracking software (Stoelting Co., Wood Dale, IL).

### Tissue Collection and Processing

Animals were euthanized on experimental day 12 via live decapitation. Brains were removed and divided for multiple analyses. Following sagittal bisection along the midline, the anterior left hemisphere (prefrontal cortex, anterior to the dorsal hippocampus at approximately Bregma +1.0mm) was fixed in 4% paraformaldehyde for 24h followed by cryoprotection in 20% sucrose solution for IBA-1 immunohistochemistry. The remaining brain tissue (posterior left hemisphere containing hippocampus and the entire right hemisphere) was flash-frozen on dry ice and stored at −80°C for PARP-1 immunohistochemistry, Western blots, and cytokine analyses. Tissue was coronally sectioned using a Leica CM1950 cryostat (Buffalo Grove, IL).

### Immunohistochemistry

#### PARP-1 Immunohistochemistry.

Frozen tissue sections (20μm) from the PFC were mounted on slides and fixed in cold acetone. Sections were incubated with anti-PARP-1 polyclonal antibody (Abcam, Cambridge, UK; 1:500) overnight at 4°C, followed by donkey anti-rabbit Alexa Fluor-594 secondary antibody (Thermo Scientific, Waltham, MA; 1:1000) for 2 hours. Images were captured using an EVOS FL Auto Imaging System (ThermoFisher Scientific) and analyzed with MCID Analysis Software (InterFocus Ltd). Three distinct regions were examined: layer 1 (L1) gray matter, layers 3-6 (L3-6) gray matter, and forceps minor of the corpus callosum (FMI) white matter.

#### PARP-1 Western Blots.

Western blotting was performed on prefrontal cortical gray matter punches to verify immunohistochemistry findings. Samples were homogenized in lysis buffer with protease and phosphatase inhibitors, separated on 4-20% gradient gels, and transferred to PVDF membranes. Membranes were probed with PARP-1 antibody (Cell Signaling Technology; 1:1000) and anti-rabbit IgG HRP-linked secondary antibody (1:5000). β-actin served as loading control. Bands were visualized using G:Box Automated Imaging (Syngene) and quantified with GeneTools software.

#### IBA-1 Immunohistochemistry.

Fixed tissue was sectioned (50μm) and processed as free-floating sections. Tissue was incubated with IBA-1 polyclonal antibody (Fujifilm; 1:1000) overnight at 4°C, followed by donkey anti-rabbit Alexa Fluor-488 secondary antibody (ThermoFisher Scientific; 1:1000). Images were captured using a Leica TCS SP8 confocal microscope and analyzed with Imaris software (Oxford Instruments) to assess microglial morphology. Ten parameters were analyzed: soma volume, soma area, soma sphericity, ellipticity (prolate), ellipticity (oblate), filament volume, filament area, filament length, dendrite branch points, and sholl intersections.

### Cytokine ELISAs

Ventral hippocampal tissue was collected and analyzed for proinflammatory cytokines using ELISA kits for IL-1β, TNF-α, and IL-6 (All from Biomatik, Wellington, DE, IL-1β: EKF57939, TNF-α: EKF57956, IL-6: EKF57855). Tissue was homogenized in RIPA buffer with protease and phosphatase inhibitors. The protocol provided by the vendor was strictly followed. Optical density was measured at 450nm using a Bio-Tek ELx880 microplate reader and converted to protein concentration (pg/mL) using standard curves.

### Statistical Analyses

Analyses were performed using GraphPad Prism version 10.6.1. Data were analyzed using one-way or two-way ANOVAs followed by Dunnett's post-hoc tests comparing treatment groups against the SAL/Stress group. For the sucrose preference test, a two-way (Treatment × Day) repeated measures ANOVA was employed. For microglia morphology analyses, hierarchical linear mixed-effects models were used to account for the nested structure of the data ( multiple cells per animal), with treatment group as a fixed effect and animal as a random effect. This approach accounts for non-independence of multiple measurements from the same animals. Post-hoc comparisons were conducted using Holm-Šídák’s multiple comparisons test. Outliers were determined using Grubbs' test. Statistical significance was set at p < 0.05. Results are presented as mean ± SEM.

## Results

### Sucrose Preference Test

**Figure 2.**
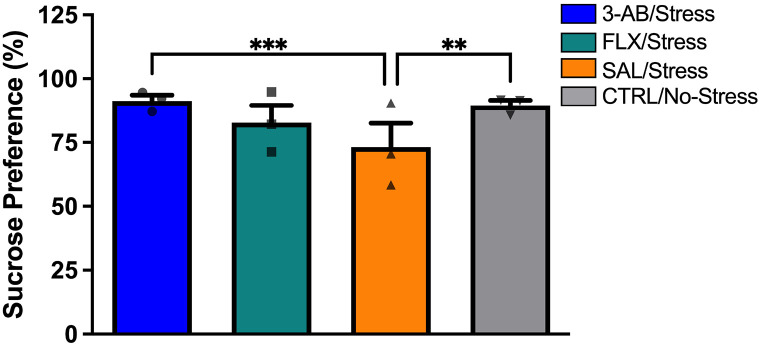
3-AB prevents stress-induced anhedonia. Sucrose preference (percent) measured on experimental days 8-10. The SAL/Stress group exhibited significantly lower preference compared to CTRL/No-Stress (*p < 0.05) and 3-AB/Stress (**p < 0.01) groups, indicating anhedonic behavior. Treatment with 3-AB prevented this stress-induced deficit. Data are mean ± SEM (n = 8 per group).

A two-way ANOVA revealed significant effects of Treatment [F(3,84) = 4.570, p = 0.005] and Day [F(2,84) = 17.505, p < 0.01] on sucrose preference, with no significant interaction. Post-hoc comparisons showed that the 3-AB/Stress group (M = 91.35 ± 5.51%) exhibited significantly higher sucrose preference than the SAL/Stress group (M = 73.16 ± 29.90%, p < 0.01). Similarly, the CTRL/No-Stress group (M = 89.57 ± 7.53%) demonstrated significantly higher sucrose preference than the SAL/Stress group (p = 0.017). The FLX/Stress group did not differ significantly from the SAL/Stress group (Fig 2).

### Social Interaction Test

**Figure 3.**
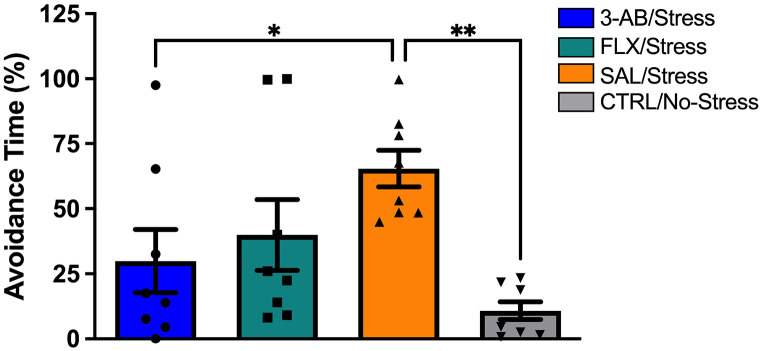
3-AB prevents stress-induced social avoidance. Time spent in the avoidance zone (percent) during the social interaction test on day 11. The SAL/Stress group exhibited significantly greater avoidance compared to 3-AB/Stress (*p < 0.05) and CTRL/No-Stress (**p < 0.01) groups. Treatment with 3-AB prevented this stress-induced social deficit. Data are mean ± SEM (n = 8 per group).

Analysis of the social interaction test revealed a significant effect of Treatment on time spent in the avoidance zone [F(3,28) = 5.263, p = 0.005]. The SAL/Stress group (M = 65.41 ± 19.92%) spent significantly more time in the avoidance zone compared to the 3-AB/Stress group (M = 29.88 ± 34.40%, p < 0.045) and the CTRL/No-Stress group (M = 10.82 ± 9.56%, p < 0.01). The FLX/Stress group (M = 39.90 ± 19.91%) did not differ significantly from the SAL/Stress group (Fig 3).

### Arterial Blood Pressure and Heart Rate Monitoring

**Figure 4.**
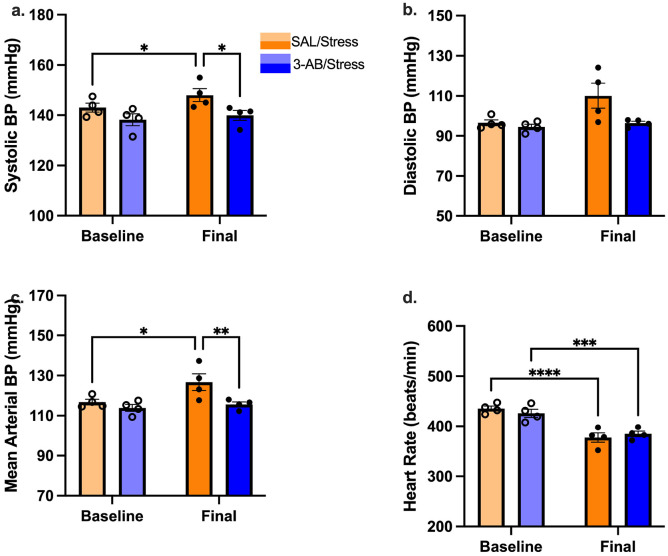
PARP-inhibition protects against stress-induced increases in arterial blood pressure. Hemodynamic parameters measured via radiotelemetry before (baseline, open circles) and after (final, filled circles) the 10-day SDS+CUS paradigm. (A) Systolic blood pressure, (B) diastolic blood pressure, (C) mean arterial pressure, and (D) heart rate. Chronic stress significantly increased systolic BP, diastolic BP, and MAP in SAL/Stress rats (* p < 0.05 vs. baseline), while 3-AB treatment prevented these elevations. SAL/Stress rats also showed significantly higher systolic BP, diastolic BP, and MAP than 3-AB/Stress rats post-stress (*p < 0.05, **p < 0.01). Heart rate decreased in both stress groups (***p < 0.001). Individual data points represent individual animals. Data are mean ± SEM (n = 4 per group).

**Figure 5.**
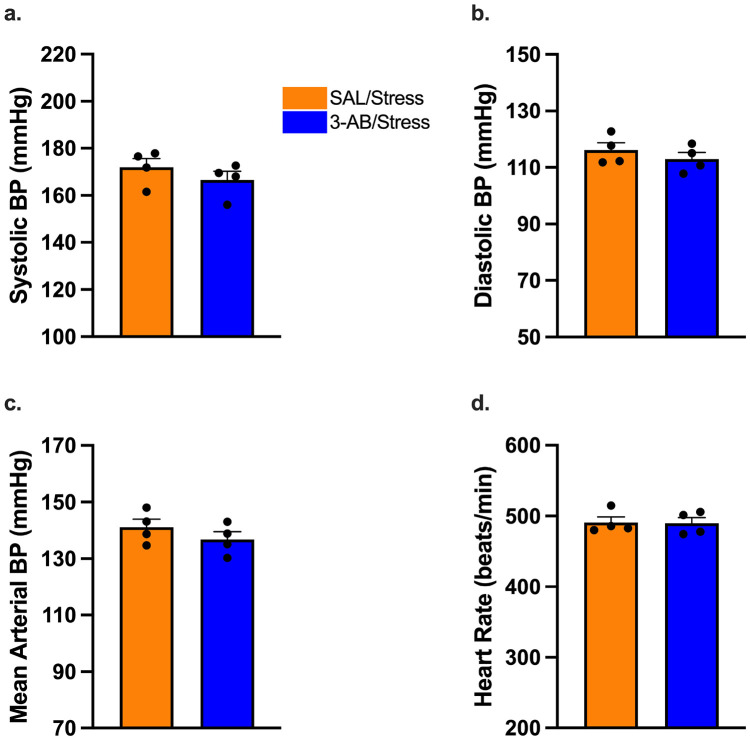
Acute hemodynamic responses during social defeat stress are similar between treatment groups. Real-time cardiovascular parameters measured via radiotelemetry during the 5-min social defeat stress sessions. (A) Systolic blood pressure, (B) diastolic blood pressure, (C) mean arterial pressure, and (D) heart rate during social defeat exposure. No significant differences were observed between SAL/Stress (orange) and 3-AB/Stress (blue) groups in any hemodynamic parameter during acute stress exposure. Individual data points represent individual animals. Data are mean ± SEM (n = 4 per group).

To assess cardiovascular effects of chronic stress and potential protective effects of 3-AB, arterial blood pressure and heart rate were monitored before, during, and after the stress paradigm (Fig 4 & 5). Baseline hemodynamic parameters during the 3-day pre-stress period showed no significant differences between groups (Fig 4).

#### Systolic Blood Pressure.

Two-way repeated measures ANOVA revealed a significant main effect of Time [F(1,6) = 9.68, p = 0.021], (Fig 4a), but no significant main effect of Treatment or Treatment × Time interaction effects (both p > 0.05). Within-group analysis showed that chronic stress significantly increased systolic blood pressure in SAL/Stress rats [baseline: 143.00 ± 1.77 vs. post-stress: 148.05 ± 2.60 mmHg; t(6) = 3.28, p = 0.017], while the 3-AB/Stress rats showed no significant change from baseline (baseline: 138.2 ± 2.4mmHg vs. final: 140.0 ± 1.9mmHg; p > 0.05). Between-group comparison revealed that 3-AB/Stress rats had significantly lower final systolic blood pressure compared to SAL/Stress rats (p = 0.020; Fig 4a), indicating that PARP inhibition prevented stress-induced hypertension.

#### Diastolic Blood Pressure.

Two-way repeated measures ANOVA revealed no significant main effects of Treatment [F(1, 6) = 5.605, p = 0.056] or Time [F(1, 6) = 5.325, p = 0.061] and no Treatment x Time interaction [F (1,6) = 3.028, p = 0.133; Fig. 4b]. Although SAL/Stress rats showed a numerical increase (baseline: 96.5 ± 1.5mmHg vs. final 110.1 ± 6.3mmHg) while 3-AB/Stress animals remained stable (baseline: 94.5 ± 1.3mmHg vs. final 96.4 ± 0.9mmHg), these differences did not reach statistical significance (Figure 4b).

#### Mean Arterial Pressure.

Analysis of mean arterial pressure showed a significant main effect of group [F(1,6) = 6.91, p = 0.039] and time [F(1,6) = 6.80, p = 0.040, Fig 4c]. SAL/Stress rats' MAP significantly increased [t(6) = 3.137, p = 0.020] after the 10 days of SDS+CUS. Additionally, SAL/Stress rats' post-stress MAP recording was significantly higher than 3-AB/Stress rats [t(12) = 3.188, p = 0.008].

#### Heart Rate.

Analysis of heart rate demonstrated a significant main effect of Time [F(1,6) = 233.4, p < 0.0001] and a significant interaction effect of Treatment × Time [F(1,6) = 6.484, p = 0.044, Figure 3d]. Post-hoc analysis using Fisher's LSD showed that the SAL/Stress rats [t(6) = 12.60, p < 0.0001] and 3-AB/Stress rats [t(6) = 9.30, p = 0.0001] showed significant decrease in heart rate after 10 days of SDS+CUS.

#### Acute Response to Social Defeat.

Hemodynamics were recorded during social defeat. There were no significant differences between the SAL/Stress and 3-AB animals in systolic blood pressure, diastolic blood pressure, MAP, or heart rate (Fig 5a-d).

### PARP-1 Expression in Rat Brain Tissue

#### Prefrontal Cortical Gray Matter (L1 and L3-L6).

**Figure 6.**
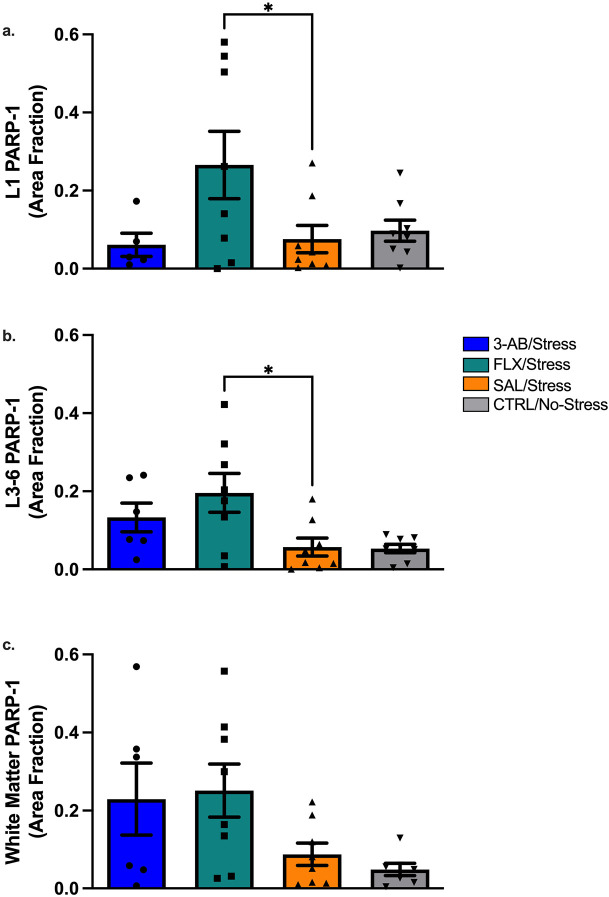
3-AB normalized PARP-1 expression while FLX increases PARP-1 expression in prefrontal cortical gray matter. PARP-1 immunoreactivity (area fraction) in prefrontal cortex subregions. (A) Layer 1 (L1) gray matter, (B) Layers 3-6 (L3-6) gray matter, and (C) forceps minor of the corpus callosum (white matter). FLX/Stress rats exhibited significantly elevated PARP-1 expression compared to SAL/Stress rats in both L1 (*p = 0.043) and L3-6 (*p = 0.012) gray matter regions. No significant differences were observed in white matter. Individual data points represent individual animals. Data are mean ± SEM (n = 8 per group).

PARP-1 immunohistochemistry revealed significant differences in PARP-1 expression in both L1 [F(3,25) = 3.11, p = 0.045] and L3-L6 [F(3,26) = 4.446, p = 0.012] regions of prefrontal cortical gray matter with the FLX/Stress group showed significantly higher PARP-1 expression in both regions compared to the SAL/Stress group (L1: p = 0.043; L3-6: p = 0.012; Fig 6a-b, respectively).

#### Forceps Minor of the Corpus Callosum.

In the prefrontal cortical white matter (FMI), a significant main effect of Treatment was observed [F(3,25) = 3.24, p = 0.039], but post-hoc comparisons using Dunnett's test revealed no significant differences between treatment groups and the SAL/Stress group (Fig 6c).

### Microglial Morphology

**Figure 7.**
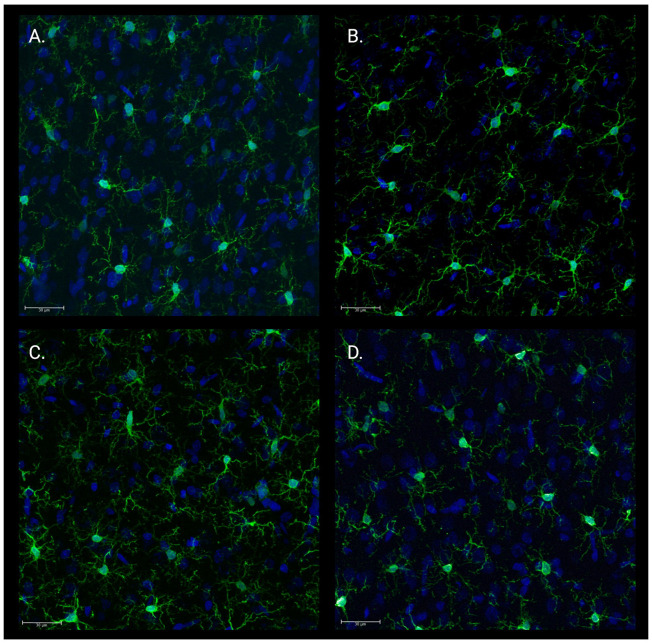
Microglial morphology in prefrontal cortex varies by treatment. Microglia (green, Iba1) and nuclei (blue, DAPI) in prefrontal cortex from (A) 3-AB/Stress, (B) FLX/Stress, (C) SAL/Stress, (D) CTRL/No-Stress groups. Morphological changes that can be observed across treatment groups, with SAL/Stress animals displaying more activated, prolate microglial phenotypes characterized by retracted processes and irregularly shaped soma compared to the ramified morphology observed in 3-AB/Stress, FLX/Stress, and CTRL/No-Stress groups. Scale bar = 30μm.

**Figure 8.**
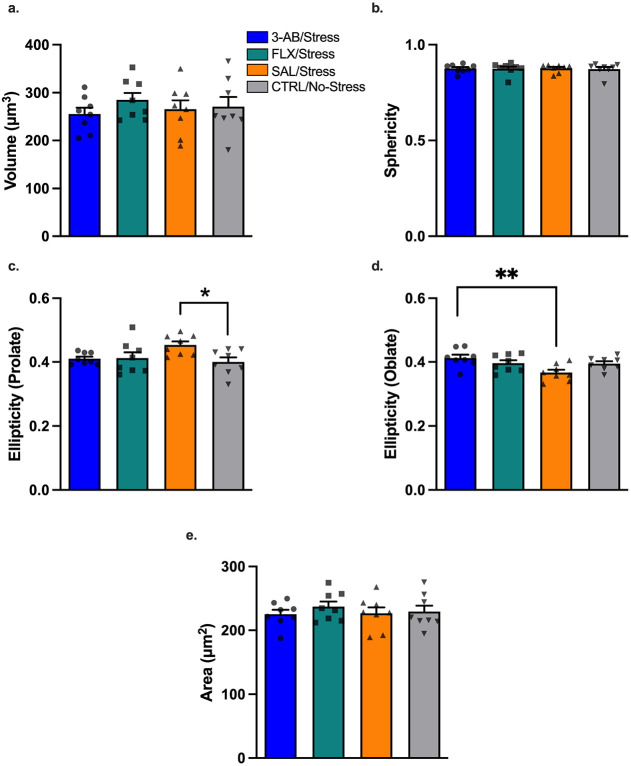
Microglial soma morphology in prefrontal cortex gray matter. Microglial soma morphology parameters from 3D reconstruction of IBA-1-immunolabled microglia (15 cells per animal). (A) Soma volume, (B) soma sphericity, (C) soma ellipticity (prolate), (D) soma ellipticity (oblate), and (E) soma area. Chronic stress induced a more prolate (elongated) microglial soma shape in SAL/Stress rats compared to CTRL/No-Stress(*p = 0.043), consistent with microglial activation. Treatment with 3-AB prevented this stress-induced morphological change, with 3-AB/Stress microglia exhibiting significantly more oblate soma than SAL/Stress (**p = 0.007). Individual data points represent individual animals. Data are mean ± SEM (n = 8 per group).

IBA-1 immunohistochemistry (Fig. 7) and 3D morphological analysis of 15 microglia per animal revealed significant differences in microglial morphology in PFC gray matter (Fig 8). Mixed-effects analysis accounting for cell clustering within animals revealed significant effects of Treatment on microglial soma shape, specifically ellipticity (prolate) [F(3,28) = 3.227, p = 0.038; Fig 8c] and ellipticity (oblate) [F(3,28) = 4.507, p = 0.011; Fig. 8d]. Post-hoc comparisons using Holm-Šídák’s multiple comparisons test showed that microglia in the CTRL/No-Stress group were significantly less prolate (M = 0.400 ± 0.031) than those in the SAL/Stress group (M = 0.453 ± 0.036, p = 0.043), consistent with a surveilling rather than activated state (Fig 8c). Additionally, 3-AB/Stress group were significantly more oblate (M = 0.413 ± 0.026) than those in the SAL/Stress group (M = 0.367 ± 0.024, p = 0.007), indicating a less activated morphology (Fig 8d). No significant differences were observed in gray matter filament morphology or white matter microglial morphology between groups (Supplementary figs S1-3).

### Cytokine Expression

Analysis of proinflammatory cytokine levels in ventral hippocampus revealed significant effects of Treatment on TNF-α [F(3,32) = 5.60, p = 0.003], IL-1β [F(3,32) = 8.356, p < 0.001], and IL-6 [F(3,30) = 4.92, p = 0.007] expression (Fig 9).

#### TNF-α.

The SAL/Stress group (M = 274.51 ± 109.59 pg/mL) exhibited significantly higher TNF-α levels compared to the 3-AB/Stress (M = 187.79 ± 37.52 pg/mL, p < 0.008), FLX/Stress (M = 167.68 ± 32.78 pg/mL, p < 0.008), and CTRL/No-Stress (M = 179.12 ± 32.12 pg/mL, p < 0.008) groups (Fig 9*a*).

#### IL-1β.

Similarly, the SAL/Stress group (M = 48.13 ± 11.77 pg/mL) showed significantly elevated IL-1β levels compared to FLX/Stress (M = 34.25 ± 8.46 pg/mL, p < 0.002), and CTRL/No-Stress (M = 32.94 ± 2.71 pg/mL, p < 0.002) groups (Fig 9*b*).

#### IL-6.

The SAL/Stress group (M = 11.50 ± 5.45 pg/mL) demonstrated significantly higher IL-6 levels compared to the FLX/Stress (M = 5.45 ± 2.18 pg/mL) and CTRL/No-Stress (M = 3.81 ± 3.55 pg/mL, p = 0.001) groups but did not significantly differ from the 3-AB/Stress group (Fig 9*c*).

## Discussion

The present study investigated the antidepressant effects of the PARP inhibitor 3-AB in a rodent model of chronic psychological stress while elucidating the neurobiological mechanisms underlying these effects. Our findings demonstrate that 3-AB produces robust antidepressant-like behavioral effects while attenuating neuroinflammatory responses to chronic stress. Although the precise molecular mechanisms require further elucidation, particularly given the absence of significant PARP-1 upregulation in stressed animals, these results suggest that PARP-1 inhibition or related downstream signaling pathways represent promising therapeutic targets for stress-related psychiatric disorders.

### Behavioral Effects of 3-AB

The sucrose preference test, a well-established measure of anhedonia in rodents^26^, revealed that 3-AB treatment prevented stress-induced anhedonic behavior, with 3-AB-treated stressed rats showing sucrose preference comparable to non-stressed controls. Similarly, in the social interaction test, 3-AB treatment prevented the development of social avoidance behavior in stressed rats. These findings corroborate previous work from our laboratory demonstrating antidepressant-like effects of 3-AB in rodent models^24^.

Interestingly, while 3-AB consistently prevented stress-induced behavioral deficits, FLX treatment failed to produce significant improvements in either sucrose preference or social interaction. This finding is contrary to our previous work^24^ and suggests possible treatment resistance in the FLX/Stress group, a phenomenon also observed in clinical populations^4,27^. This differential response highlights the potential value of targeting alternative mechanisms, such as PARP inhibition, for treatment-resistant depression.

### Chronic Stress-Induced Hypertension and 3-AB Protection

Our cardiovascular monitoring revealed that chronic stress significantly elevated systolic blood pressure and mean arterial pressure in saline-treated rats, while 3-AB treatment prevented these stress-induced hemodynamic changes. These findings extend previous observations linking chronic psychological stress to cardiovascular dysfunction^16-18^ and demonstrate that PARP inhibition provides protection across multiple physiological systems. The cardiovascular protective effects of 3-AB can be at least partially attributed to preservation of autonomic nervous system function. PARP-1 deletion has been shown to restore baroreflex sensitivity and improve autonomic responses in dyslipidemic mice without affecting baseline heart rate or blood pressure^28^, a pattern similar to our findings. The mechanism appears to involve PARP-1-mediated oxidative stress, which impairs baroreflex function through inducible nitric oxide (iNOS) upregulation in cardiovascular regulatory pathways^28^. Additionally, PARP-1 overactivation has been implicated in endothelial dysfunction and vascular inflammation^29,30^. Importantly, both groups showed similar acute hemodynamic responses during social defeat sessions (Fig. 5), indicating that 3-AB did not impede on normal autonomic reactivity to acute stressors. The key difference lies in the recovery, 3-AB-treated animals returned to baseline hemodynamics following stress cessation, while saline-treated animals developed persistent cardiovascular perturbations. This pattern suggest that PARP inhibition preserves adaptive stress responses while preventing the maladaptive cardiovascular remodeling associated with chronic stress.

### PARP-1 Expression and Fluoxetine Interaction

Recent research has identified cytotoxic effects of SSRIs, including fluoxetine, on astrocytes^31^. It is thought that fluoxetine may directly bind to the GluR1 subunit of AMPA receptors, triggering intracellular calcium influx that can lead to increased cytotoxic activity in astrocytes^31^. This mechanism might explain the elevated PARP-1 expression observed in FLX-treated rats in our study. The dissociation between PARP-1 immunohistochemistry and Western blot results may reflect methodological differences in sensitivity or regional specificity, as immunohistochemistry can detect cellular and subcellular localization patterns not captured by whole-tissue homogenates.

In addition, PARP-1 levels in the SAL/Stress group did not significantly differ from the 3-AB/Stress group, contrary to our hypothesis. This may reflect the temporal dynamics of stress-induced PARP-1 regulation in the prefrontal cortex. Our tissue collection timepoint (48h post-stressor) may have preceded peak PARP-1 expression, as stress-induced molecular changes exhibit variable temporal profiles across brain regions. Future studies should examine multiple post-stress timepoints (24-72h) to characterize the temporal trajectory of PARP-1 expression and assess enzymatic activity in addition to protein levels, as catalytic function may change independent of total protein abundance.

### Microglial Activation and Neuroinflammation

Our analysis of microglial morphology revealed that stressed rats treated with saline exhibited significantly more prolate (elongated) microglia in PFC gray matter compared to non-stressed controls, consistent with early microglial activation^32^. In contrast, stressed rats treated with 3-AB displayed significantly more oblate microglia, resembling the non-activated morphology seen in control rats^32^. These findings suggest that 3-AB may exert its antidepressant effects in part by preventing stress-induced microglial activation.

The lack of significant morphological changes in white matter microglia was unexpected, given our previous finding of increased oxidative DNA damage in PFC white matter following chronic stress^21,24^. This discrepancy may be due to the timing of tissue collection (48 h after the final stressor), allowing microglia to return to a surveilling state. Future studies should examine microglial activation at various time points following stress exposure to capture the temporal dynamics of microglial responses to chronic stress.

### Proinflammatory Cytokine Expression

Analysis of proinflammatory cytokines in the ventral hippocampus revealed that 3-AB treatment significantly attenuated stress-induced increases in TNF-α and IL-1β levels. These findings are consistent with previous research demonstrating anti-inflammatory effects of PARP inhibitors^10^ and provide a potential mechanism for 3-AB's antidepressant effects. Proinflammatory cytokines have been implicated in the pathophysiology of depression through various mechanisms, including disruption of monoamine neurotransmission^33^, activation of the kynurenine pathway^34^, and promotion of oxidative stress^11^. By attenuating stress-induced increases in proinflammatory cytokines, 3-AB may prevent these detrimental effects on neuronal function and mood regulation.

### Proposed Mechanism and Clinical Implications

Based on our findings, we propose that 3-AB exerts antidepressant effects primarily through inhibition of PARP-1-mediated neuroinflammatory processes. By preventing PARP-1 activation, 3-AB may reduce NF-κB-dependent transcription of proinflammatory genes, thereby decreasing microglial activation and proinflammatory cytokine production. This anti-inflammatory action would help maintain normal neuronal function and prevent stress-induced behavioral deficits. The prevention of stress-induced hypertension by PARP inhibition indicates that it provides multi-system benefits that may be particularly valuable in treating the complex pathophysiology of stress-related disorders and increased risk of cardiovascular disease in this population. Exploratory analyses of gut microbiome composition revealed stress-induced alterations that were partially prevented by 3-AB treatment, further supporting multi-system effects (See Supplemental Materials).

These findings have important clinical implications. PARP inhibitors are already approved for cancer treatment, facilitating potential repurposing for psychiatric indications. Our data suggest that PARP inhibitors may be particularly valuable for treatment-resistant depression, which affects a substantial proportion of MDD patients. Additionally, the case report from our laboratory demonstrating rapid antidepressant actions of the PARP inhibitor niraparib in a patient with treatment-resistant depression^25^ further supports the translational potential of this approach.

### Exploratory Microbiome Findings

Although not the primary focus of this study, exploratory microbiome analyses were conducted to assess potential peripheral effects of chronic stress and PARP inhibition (see Supplemental Materials). PERMANOVA analysis of 16S rRNA sequencing data revealed no differences in overall microbial beta diversity among groups. However, *Bacteroides dorei* abundance was significantly reduced in SAL/Stress rats compared with both the CTRL/No-Stress group (p < 0.005) and the 3-AB/Stress group (p < 0.001), while no difference was observed between the CTRL/No-Stress and 3-AB/Stress groups. Members of the *Bacteroides* genus promote gut health through protective measures, including modulation of detoxifying peroxidases as part of the antioxidant response^35^. These pathways are particularly relevant given the antioxidant deficits we have demonstrated in human stress-related disorders^21,36,37^. Although relatively understudied compared to other *Bacteroides* species, *B. dorei* has been shown to modulate immune responses in mice^38^.

Analysis of microbial metabolites revealed a significant reduction in valeric acid levels in SAL/Stress animals compared with CTRL/No-Stress animals (p < 0.05; Supplemental Fig. 4f), while 3-AB-treated animals showed intermediate levels that did not differ significantly from either group. Decreases in valeric acid have been reported in mice exposed to chronic unpredictable mild stress^39^ and short-chain fatty acid supplementation has been shown to improve behavioral deficits in stressed mice^40^. These exploratory findings, while limited by the single post-stress timepoint design, suggest that chronic stress induces peripheral gut alterations that may be prevented by PARP inhibition. Taken together with our cardiovascular data, these results suggest that stress and treatment with 3-AB may influence additional multi-system physiological changes such as, gut microbiota and its metabolic outputs, potentially leading to behavioral improvements. Microbial measures may therefore serve as useful biomarkers in future studies evaluating the clinical utility of PARP inhibitors for stress-related disorders and thus warrant further studies.

### Future Directions

Several important questions remain to be addressed in future research. First, our study focused exclusively on male rats, despite the higher prevalence of MDD in women. Sex differences in stress responses, neuroinflammatory processes, and PARP-1 signaling are well-documented^41,42^, and future studies should include female rats to assess potential sex-specific effects of PARP inhibition. Second, the timing of tissue collection (48h after the final stressor) may have allowed for partial resolution of neuroinflammatory responses, potentially obscuring some acute effects. Time-course studies examining neuroinflammatory markers at multiple time points following stress exposure would provide valuable insights into the temporal dynamics of stress-induced pathophysiology and PARP inhibitor effects. Third, future research should investigate the long-term effects of PARP inhibitor treatment, potential interactions with traditional antidepressants, and effects on other neurobiological systems implicated in depression, such as neurotrophic signaling and neurogenesis. Additionally, the unexpected finding of elevated PARP-1 expression in FLX/stress-treated rats warrants further investigation into the relationship between serotonergic signaling and PARP-1 activity.

### Conclusion

This study provides compelling evidence for the antidepressant-like effects of the PARP inhibitor 3-AB in a rodent model of chronic psychological stress. Our findings demonstrate that 3-AB prevents stress-induced behavioral deficits by attenuating neuroinflammatory responses, including microglial activation and proinflammatory cytokine production, while also providing cardiovascular protection. These results support the growing recognition of neuroinflammation as a key pathophysiological process in stress-related disorders and highlight PARP inhibition as a promising novel approach for treating depression, particularly in cases resistant to conventional therapies. The multi-system benefits of PARP inhibition, including behavioral, neuroinflammatory, and cardiovascular protection, suggest that this approach may be particularly valuable for addressing the complex and interconnected pathophysiology of treatment-resistant depression. Future clinical studies should investigate the efficacy and safety of PARP inhibitors in patients with treatment-resistant depression.

## Supplementary Material

Supplement 1

**Note:** Supplemental materials include microglial morphology graphs for gray matter filaments, white matter soma and filaments, exploratory analyses of gut microbiome composition (16S rRNA sequencing), short-chain fatty acid quantification, with detailed methods and results available online.

## Figures and Tables

**Figure 1.**
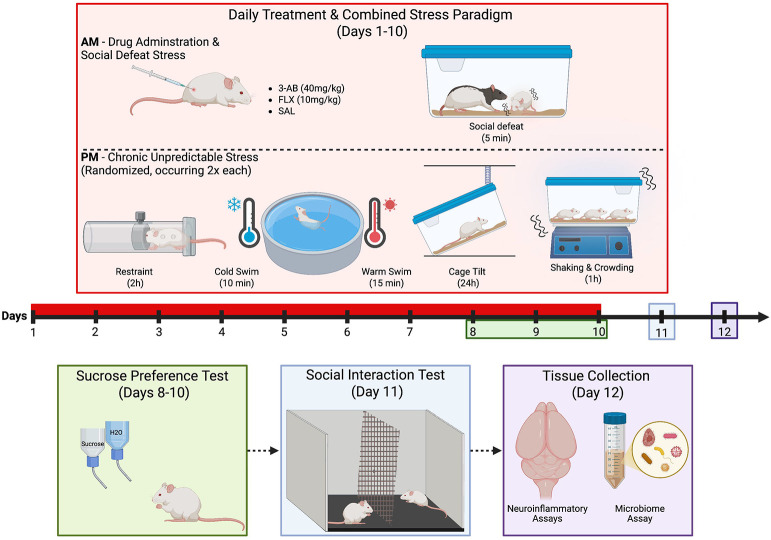
Experimental timeline and Behavioral Design. Adult Male Sprague-Dawley rats (n = 8/group) were exposed to 10 days of combined social defeat stress (SDS, 5min/day, AM) and chronic unpredictable stress (CUS, rotating PM stressors) while receiving daily injections of 3-AB (40mg/kg, s.c.), FLX (10 mg/kg, i.p.), or SAL (i.p.). CUS stressors included restraint stress (2h), cold swim (18°C, 10 min), warm swim (25°C, 15 min), cage tilt (24h), and shaking and crowding (1h). Anhedonia was assessed via sucrose preference testing (Days 8-10, 7PM-9PM), and social avoidance was measured on Day 11. Tissue was collected 48h post-stress (Day 12) for PARP-1 analysis (IHC), microglial morphology assessment (IHC), cytokine quantification (ELISA), and microbiome profiling (SCFA assay). Figure created using BioRender.

**Figure 9.** 3-AB and FLX prevent stress-induced increases in hippocampal proinflammatory cytokines. Hippocampal cytokine concentrations measured by ELISA. (A) Tumor necrosis factor-alpha (TNF-α), (B) interleukin-1 beta (IL-1β), and (C) interleukin-6 (IL-6). Chronic stress significantly elevated TNF-α in SAL/Stress animals compared to 3-AB/Stress, FLX/Stress, and CTRL/No-Stress (p’s < 0.05). For IL-1β and IL-6, Sal/Stress was significantly elevated compared to CTRL/No-Stress and FLX/Stress animals (p’s < 0.05). Individual data points represent individual animals. Data are mean ± SEM (n = 8 per group).

## Data Availability

The data that support the findings of this study are available from the corresponding author upon reasonable request.

## Significant Outcomes

- PARP inhibition with 3-aminobenzamide (3-AB) prevented stress-induced behavioral deficits (anhedonia and social avoidance) in a validated rodent model combining social defeat and chronic unpredictable stress.
- 3-AB treatment prevented stress-induced increases in arterial blood pressure.
- PARP inhibition prevented microglial activation and reduced proinflammatory cytokine expression (IL-1β and TNF-α) in stress-sensitive brain regions, supporting an anti-neuroinflammatory mechanism of action.
- FLX-treated stressed rats unexpectedly showed elevated PARP-1 expression in prefrontal cortical gray matter, suggesting a previously unrecognized interaction between serotonergic antidepressants and PARP-1 signaling that warrants further investigation.
- The multi-system protective effects of PARP inhibition, spanning behavioral, cardiovascular, and neuroinflammatory domains, suggest therapeutic potential for treatment-resistant depression.

## Limitations

- This study examined only male rats, limiting generalizability to female subjects despite the higher prevalence of MDD in women.
- Behavioral assessments were limited to anhedonia and social interaction; additional tests of other depression-relevant behaviors would provide a more comprehensive phenotypic profile.
- Long-term effects of 3-AB treatment beyond the 10-day stress paradigm remain unexplored.
