# Supplementary material for: PARP Inhibition with 3-Aminobenzimide Attenuates Behavioral, Cardiovascular, and Neuroinflammatory Effects of Chronic Stress
Source: bioRxiv. 2026 May 1:2026.04.28.721400. Preprint. [Version 1] doi: 10.64898/2026.04.28.721400 (PMC13142347; doi:10.64898/2026.04.28.721400)
Supplement: Supplement 1 [file media-1.pdf]

Supplementary Material

Supplemental Methods for Microbiome Analysis

DNA purification and microbiome sequencing

Feces were obtained from rats just before euthanasia in clean, dry cages, and also taken from the colon after euthanasia. It was stored at ultralow temperatures, -80°C until use. Approximately 100 mg of tissue was used with the PowerSoil Pro DNA extraction kit (Qiagen; A7014, Germantown, MD) according to the manufacturer's instructions. In brief, the tissue was exposed to bead-based homogenization followed by membrane binding, multiple wash steps, and elution via centrifugation. DNA from the samples was qualified and quantified via ThermoScientific™ NanoDrop™ UV-Vis Spectrophotometer. Extracted DNA was standardized to 20 ng/ul and transferred to the East Tennessee State University Molecular Biology Core Facility for library construction. Primers flanking the V3 and V4 domains of the bacterial 16S rRNA gene were used to amplify the DNA using the 16S Metagenomic Sequencing Library Preparation System (Illumina; San Diego, CA) following the manufacturer's instructions. Sequencing was performed by the University of Tennessee Genomics Center using the Illumina MiSeq machine. All sample sequencing was performed with the same flow cell.

Microbiome analysis

Data analysis for the sequences was performed using the Qiagen CLC Genomics Workbench, according to the manufacturer's instructions provided in the OTU clustering tutorial (Qiagen; Germantown, MD). First, adapter sequences were trimmed, and the resulting sequences were evaluated for high coverage with at least 100 reads present. The Silva complete database was used to cluster the high-coverage sequences into operational taxonomic units (OTUs). Additionally, OTUs with low abundance were removed from analysis. Phylogeny trees

were constructed using the OTU data with MUSCLE alignment and used for alpha and beta diversity analysis. OTUs were used to conduct a PERMANOVA analysis to determine overall diversity and construct differential abundance tables between the groups.

Short-chain fatty acid (SCFA) gas chromatography

Approximately 500 mg of feces per sample was freeze-dried using a LABCONCO flask system. Samples were exposed to -50 °C for at least 12 hours at .077 mBar of pressure. Short chain fatty acids (SCFAs) were extracted using a procedure described previously[43]. In brief, SCFA extraction solution was prepared by adding oxalic acid (0.1 mol/L) and sodium azide (40 mmol/L) to 80 mg of freeze-dried fecal samples in 16 x 100 mm glass tubes, followed by vigorous mixing. Solution was centrifuged three times with supernatant removed after each centrifugation and stored in an ultralow freezer until use. SCFA concentrations were determined using a gas chromatography machine (Shimadzu GC2010) equipped with a capillary column (Sigma Aldrich, AB-Was Plus) with a flame ionization detector. The machine reported the concentration as parts per million (ppm) for each individual compound. SCFA concentrations were normalized to 80 mg of tissue for each sample. Values were subsequently converted and reported as nmol/mg.

Statistical Analysis for SCFA

Differential expression values for the microbiome 16S sequencing data was performed using CLC Workbench OTU clustering tutorial. All statistical analyses for SCFA were performed using GraphPad V10. SCFA concentrations were initially assessed for outliers using a ROUT test. Normality for the data sets for each unique SCFA was determined using Shapiro-Wilk and Kolmogorov-Smirnov tests. A one-way ANOVA was used to assess differences

among the three groups if the control group passed normality tests. A Kruskal-Wallis test was used if the control group was not normally distributed.

Supplementary Figures

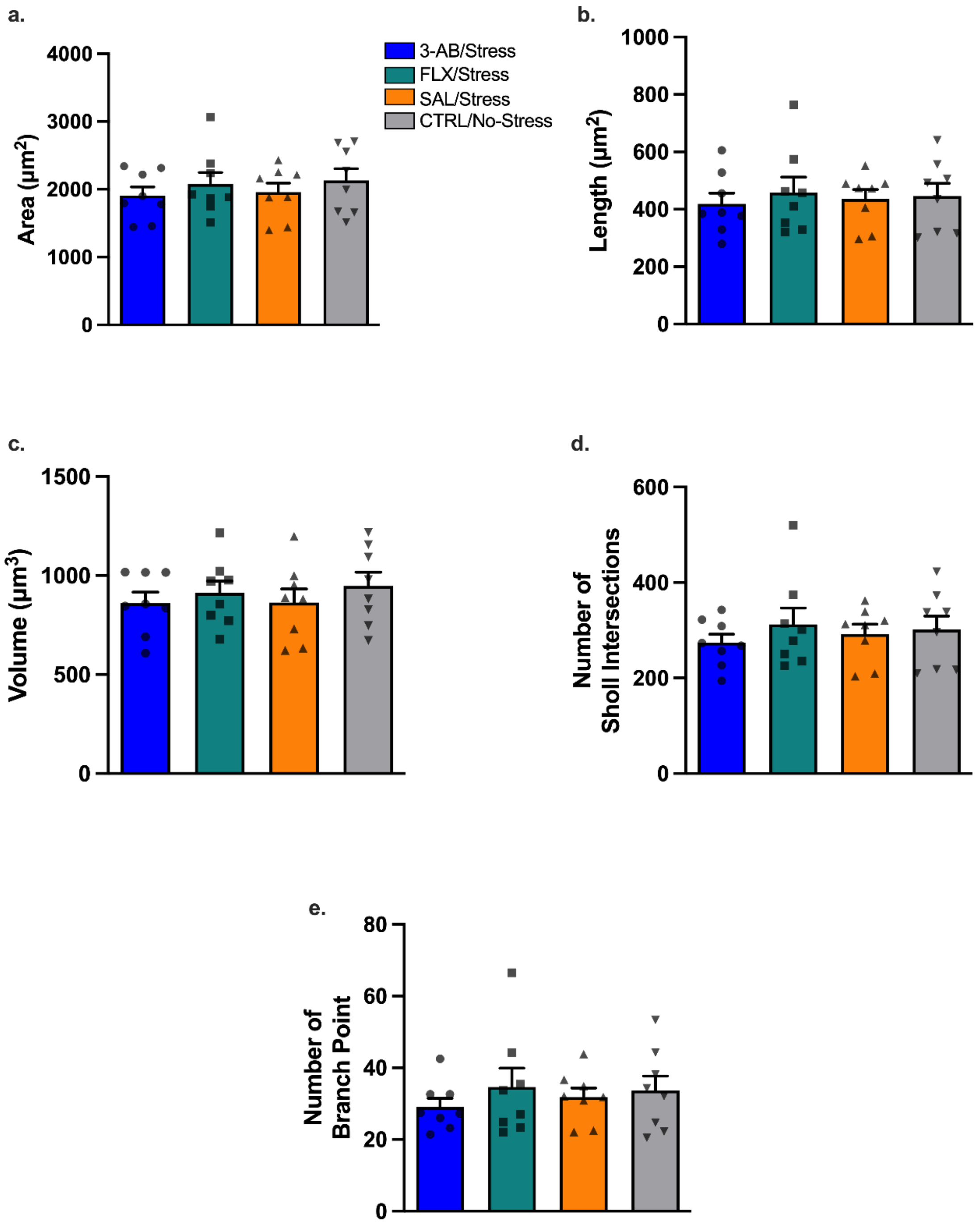

Supplementary Figure S1. Microglial filament morphology in prefrontal cortex gray matter shows no treatment-related differences. Microglial filament/ process characteristics measured from 3D reconstructed IBA-1-immunolabeled cells. (A) Filament area, (B) filament length, (C) filament volume, (D) number of sholl intersections, and (E) number of branch points. While stress significantly altered microglial soma morphology (Fig 7), no significant treatment

effects were observed on filament parameters. Individual data points represent individual animals. Data are mean ± SEM (n = 4 per group).

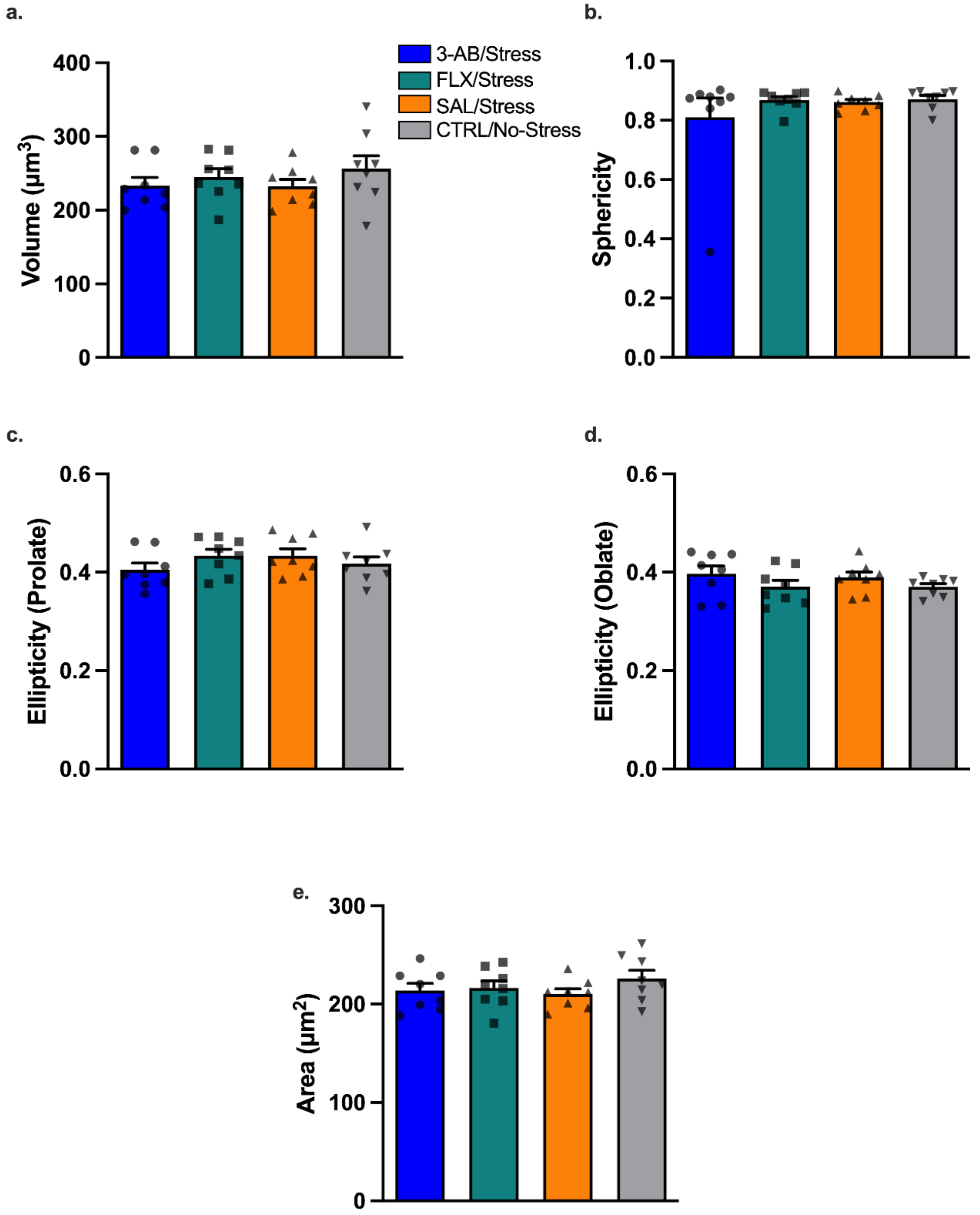

Supplemental Figure S2. Microglial soma morphology in prefrontal cortical white matter.

Microglial soma morphology parameters from 3D reconstruction of IBA-1-immunolabled

microglia (15 cells per animal). (A) Soma volume, (B) soma sphericity, (C) soma ellipticity (prolate), (D) soma ellipticity (oblate), and (E) soma area. There were no significant differences in PFC white matter microglia soma morphology. Individual data points represent individual animals. Data are mean ± SEM (n = 8 per group).

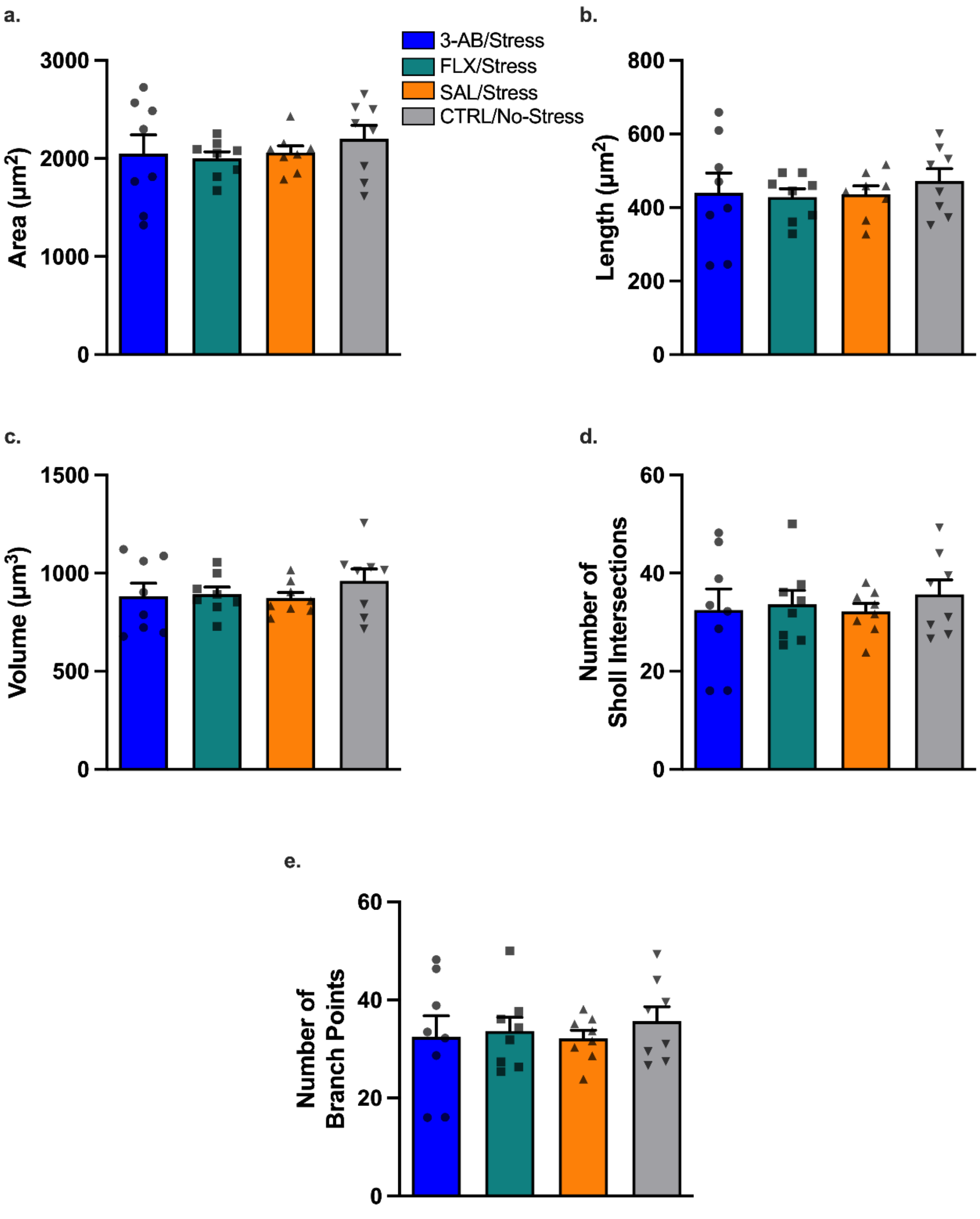

Supplemental Figure S3. White matter microglial filament morphology in prefrontal cortex. Microglial filament/ process characteristics measured from 3D reconstructed IBA-1-immunolabeled cells. (A) Filament area, (B) filament length, (C) filament volume, (D) number of sholl intersections, and (E) number of branch points. While stress significantly altered microglial soma morphology (Fig 7), no significant treatment effects were observed on filament parameters. Individual data points represent animals. Data are mean ± SEM (n = 8 per group).

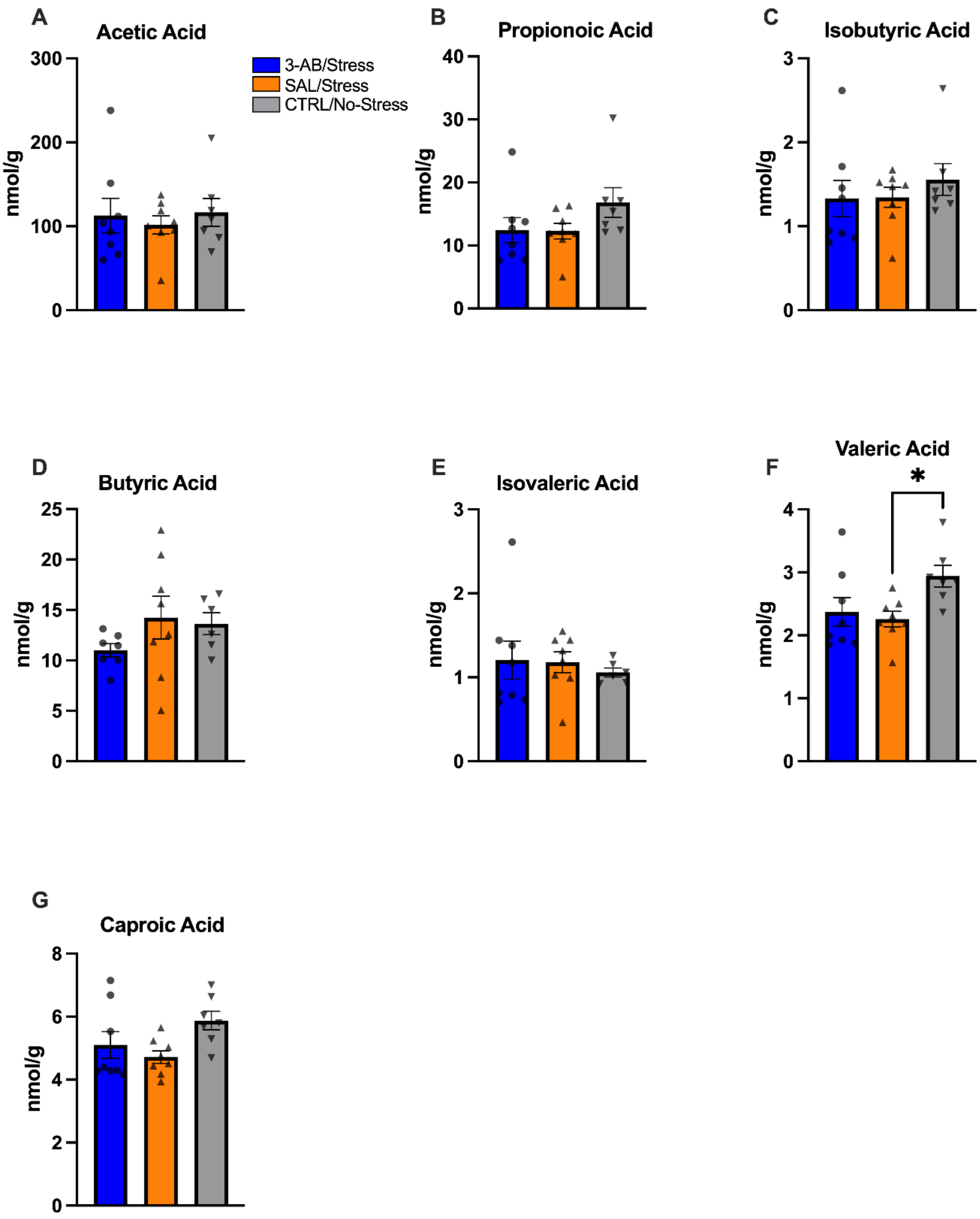

Supplemental Figure S4. Chronic stress reduces cecal valeric acid concentrations. Short-chain fatty acid (SCFA) concentrations measured by gas chromatography-mass spectrometry in cecal contents collected on day 12. (A) Acetic acid, (B) propionic acid, (C) isobutyric acid, (D) butyric acid, (E) isovaleric acid, (F) valeric acid, and (G) caproic acid. Rats in the SAL/Stress

group showed significantly lower concentrations of valeric acid compared to the CTRL/No-Stress group (* $p < 0.05$), no significant treatment effects were observed in other SCFA. Individual data points represent individual animals. Data are mean $\pm$ SEM ($n = 7$-8 per group).
